# Epithelial cell-derived microvesicles activate macrophages and promote inflammation *via* microvesicle-containing microRNAs

**DOI:** 10.1038/srep35250

**Published:** 2016-10-12

**Authors:** Heedoo Lee, Duo Zhang, Ziwen Zhu, Charles S. Dela Cruz, Yang Jin

**Affiliations:** 1 Division of Pulmonary and Critical Care Medicine, Department of Medicine, Boston University, Boston, MA 02118, USA; 2 Section of Pulmonary, Critical Care and Sleep Medicine, Yale University School of Medicine, New Haven, CT 06519, USA.

## Abstract

Intercellular communications between lung epithelial cells and alveolar macrophages play an essential role in host defense against acute lung injury. Hyperoxia-induced oxidative stress is an established model to mimic human lung injury. We show that after hyperoxia-associated oxidative stress, a large amount of extracellular vesicles (EVs) are detectable in bronchoalveolar lavage fluid (BALF) and culture medium of lung epithelial cells. Microvesicles (MVs), but not exosomes (Exos) or apoptotic bodies (Abs), are the main type of EVs found in the early stages after hyperoxia. Among all the MV compositions, small RNAs are altered the most significantly after hyperoxia-associated oxidative stress. We further confirmed that hyperoxia up-regulates the levels of certain specific miRNAs in the epithelial cell-derived MVs, such as the miR-320a and miR-221. Functionally, the hyperoxia-induced epithelial MVs promote macrophage activation *in vitro* and facilitate the recruitment of immunomodulatory cells *in vivo* detected in BALF. Using MV as a cargo, delivery of the specific miRNA-enriched epithelial MVs (miR-221 and/or miR-320a) also triggers macrophage-mediated pro-inflammatory effects. Collectively, epithelial cell-derived MVs promote macrophage-regulated lung inflammatory responses *via* MV-shuttling miRNAs.

In the past decade, emerging evidence suggests that extracellular vesicles (EVs) play an essential role in intercellular cross-talks^1^,^2^. Accumulating reports have demonstrated that EV-shuttling molecules, including proteins, RNAs, microRNAs (miRNAs) and lipids, potentially exerting crucial physiological and pathological functions in tumorigenesis^3^ and metastasis^4^. These novel discoveries shed light on the development of novel tumor markers and new therapies^5^,^6^. However, less studies focus on the pathogenesis of non-cancer-related diseases. For example, very little is known on the role of EVs in lung diseases.

Acute respiratory distress syndrome (ARDS) is a devastating disease featured with profound lung injury and characterized by the disruption of the epithelial and endothelial barrier, flooding of the alveolar compartment with protein-rich fluids, and recruitment of neutrophils into the alveolar space^7^,^8^. Numerous types of cells reside in the lungs and how they communicate with each other is largely understood. Human lung epithelium has a large surface area and epithelial cells play a key role in innate immunity against noxious insults and are involved in the recruitment of various inflammatory mediators into the alveolae^9^,^10^. These epithelial cells are important in the pathogenesis and resolution of acute lung injury (ALI)^11^,^12^. Epithelial cells respond to sterile injury and infection and activate alveolar macrophages^13^,^14^,^15^. Activated alveolar macrophages, along with other lung cells (which ones?), regulate leukocyte influx through the production of proinflammatory cytokines, chemokines and other factors that modulate chemokine gradients and effect leukocyte migration^16^,^17^,^18^. However, how epithelial cells communicate with alveolar macrophages remains unclear. Despite that EVs have been thought as potential candidates in mediating cell-cell crosstalks in cancer metastasis and other disease processes^19^,^20^, not much is known about the role of EVs in the lung. Currently, the EV generations in the lungs, the release of EVs from lung cells, and the functions of lung-derived EVs are poorly delineated, impeding the development of novel targets for novel diagnosis and therapy of lung injury.

The contents, size and membrane composition of EVs are heterogeneous based on the cellular sources and environmental stimuli^14^,^21^,^22^. According to the International Society of Extracellular Vesicles, three main subgroups of EVs have been defined based on the size, the membrane compositions and the mechanisms of formation^23^. Apoptotic bodies (ABs) (500–2000 mm diameter) are formed by plasma membrane blebbing in the process of undergoing apoptosis. Microvesicles (MVs) (50–500 nm) are the second subgroup, comprising of different sized vesicles directly protruding from plasma membranes. Exosomes (Exos) are the smallest subgroup (approximately 30–100 nm in diameter) and are released from cells after multivesicular bodies (MVBs) fuse with the plasma membrane^20^. A subset of proteins derived from parent cells are often detectable in EVs. Surfactant proteins (SPs) can be detected in the EVs derived from lung epithelial cells^24^. Interestingly, MVs and exosomes also carry distinct proteins that can be found only in one of the two. For instance, vesicle-associated membrane protein 3 (VAMP3) can be found in the MVs generated from melanoma cells, while transferrin receptors are highly enriched in exosomes, but missing in the MVs^25^. However, no single marker can uniquely identify each subgroup of EVs. Proteins which often have been used as markers of EVs but not specific to either exosomes or MVs, include: tetraspanins such as CD9, CD63, CD81 and CD82^26^.

EV-containing RNAs were first described in EVs derived from murine stem cells^27^. Despite the smaller sizes of EV intact messenger RNAs (mRNAs), long non-coding RNAs (lncRNAs), ribosomal RNA (rRNA) and the fragments of these intact RNA molecules have all been identified in EVs^23^,^28^,^29^. MiRNAs are 20–22 nt small non-coding RNA molecules which exert essential regulatory functions on their target genes^30^. Many types of miRNAs have been identified in EVs^31^,^32^,^33^. However, detailed characterizations on the miRNA profiles in exosomes or MVs require further investigation, particular in non-tumor cells. Recent studies have indicated that the number of copies of “highly upregulated” miRNAs found in tumor cells is in fact very low in each individual exosome detected in plasma^34^. This raises the question of whether these exosomal miRNAs indeed can have physiological or pathological effects on their target cells^35^. That said, no studies have explored the miRNAs contained in MVs. Therefore, before performing any further investigations that is focused on the role of EV-miRNAs, we need to first determine the type of EVs that is generated in lungs after noxious insults and determine whether these stimuli-induced EVs contain miRNAs. In this study, we will characterize the generation of EVs, their category/type based on size, composition and function in lung epithelial cells, as well as whether EVs play a role in the crosstalk between epithelial cells and alveolar macrophages.

## Results

### Characterization of the EV generation and release in lung BALF and epithelial cells

**Figure 1:**
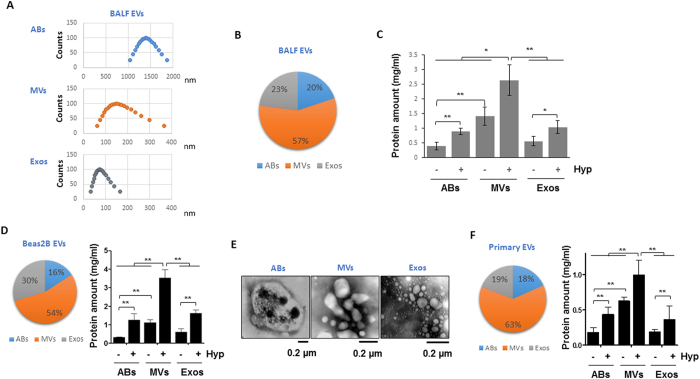
Characterization of the extracellular vesicles (EVs) generated from BALF and lung epithelial cells. (**A,B**) Three types of EVs were isolated from mouse broncho-alveolar lavage fluid (BALF), including apoptotic bodies (ABs), microvesicles (MVs), and exosomes (Exos). The sizes of the isolated EVs were measured using Dynamic Light Scattering (DLS) (**A**). Pie graph indicates the percentages of each type of EVs derived from BALF (**B**). (**C**) Mice were exposed to hyperoxia for 3 days, followed by isolation of three types of EVs from BALF. Amount of EVs were shown in the bar graph. n = 4 mice per group. Bease2B cells (**D**) and primary epithelial cells (**F**) were exposed to hyperoxia for 2 days, followed by isolation of EVs. Left panels show pie graphs indicating the percentages of each type of EVs. Right panels show the protein amounts of EVs in bar graphs. (**E**) Three types of EVs were isolated from Beas2B cells, and visualized using transmission electron microscope (TEM). Data represent mean ± SD of three independent experiments with identical results.

We first determined the generation and release of EVs in mouse lungs. We obtained broncho-alveolar lavage fluid (BALF) and isolated EVs using ultracentrifugation as previously described^23^. Based on the sizes of EVs that were determined using DLS (spell out as well), we identified three types of EVs and the range of sizes as shown in Fig. 1A. The percentages of the three types of EVs are illustrated in Fig. 1B. Of all the EVs, 57% were MVs, 20% were ABs and 23% were Exos (Fig. 1B). Hyperoxia significantly augmented the levels of MVs in BALF, comparing to ABs or Exos (Fig. 1C). We previously have shown that majority of EVs are derived from lung epithelial cells after hyperoxia^14^. Therefore, to obtain a convenient cellular model to study the underlying mechanisms, we exposed Beas2B human lung epithelial cells to hyperoxia. Similarly, majority of Beas2B epithelial cell-derived EVs fell into the ranges of MVs (53%) (Fig. 1D). The ABs, MVs and Exos generated from Beas2B cells were visualized under electric microscopy (Fig. 1E). To confirm this observation, we further isolated mouse lung primary epithelial cells and exposed them to hyperoxia. Similar results were obtained. As shown in Fig. 1F, again, 63% of EVs were MVs, while 18% and 19% of EVs were ABs and Exos, respectively (Fig. 1F). Previously, we have shown that the BALF EVs are mainly derived from the epithelial cells in the presence of hyperoxia^14^.

### Characterization of the EV compositions in lung BALF and epithelial cells

**Figure 2:**
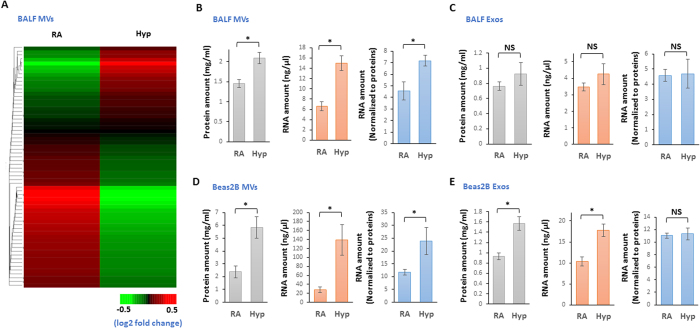
RNAs are accumulated in MVs in response to hyperoxia. (**A**) Heat map of the miRNA profiles in the MVs which were isolated from BALF. (**B–E**) Mice and Beas2B cells were exposed to hyperoxia for 2 days. MVs and Exos were isolated from BALF (**B,C**) and cultured medium of the Bease2B cells (**D,E**) followed by isolation of total RNAs from the MVs or Exos. Proteins, RNAs, and normalized RNA using protein amounts were shown. Data represent mean ± SD of three independent experiments with the similar results.

MiRNA compositions in hyperoxia-induced MVs were analyzed using microRNA arrays. Heat map is shown on Fig. 2A. Hyperoxia augmented the level of MV-protein, MV-RNA (Fig. 2B, left and middle panels). After normalization with the elevated protein, MV-RNA after hyperoxia remains significantly higher than the MV-RNA in RA (Fig. 2B, right panel). Interestingly, hyperoxia failed to alter the level of Exo-protein, Exo-RNA (Fig. 2C, left and middle panels). After normalization with Exo-protein, Exo-RNA after hyperoxia remains at the same level comparing to those obtained in RA (Fig. 2C, right panel). We next evaluate the MV-RNA and Exo-RNA using human lung epithelial cells. As shown in Fig. 2D,E, hyperoxia augmented the level of MV-protein and MV-RNA which were derived from Beas2B cells (Fig. 2D, left and middle panels). After normalization with the elevated protein, Beas2B cell-derived MV-RNA after hyperoxia remains significantly higher than MV-RNA in RA (Fig. 2D, right panel). Despite that hyperoxia augmented the level of Exo-protein and Exo-RNA which were derived from Beas2B cells (Fig. 2E, left and middle panels), after normalization with the elevated protein, Beas2B cell-derived Exo-RNA after hyperoxia remains at the same level compared to those obtained in RA (Fig. 2E, right panel). This observation is consistent with those results obtained using BALF.

### Quantification of the MV counts in the absence or presence of hyperoxia and determination of MV-RNA or MV-protein levels in each MV

**Figure 3:**
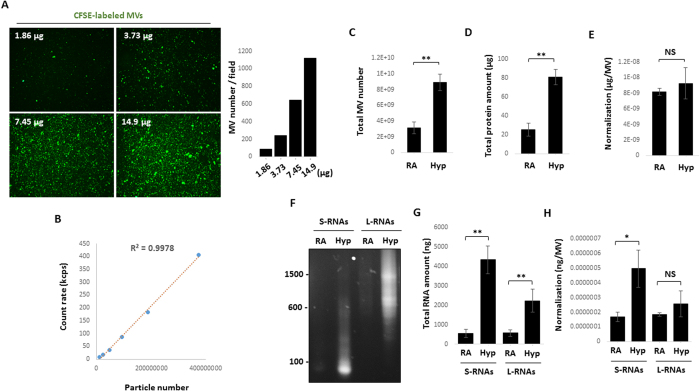
Quantification of RNA and protein amount in each MV. (**A**) MVs were isolated from Beas2B cells, and labeled with carboxyfluorescein succinimidyl ester (CFSE). Indicated protein amount of MVs were fixed and visualized using fluorescence microscope. Vesicles in field (20x magnification) were counted using NIH ImageJ software (right panel). (**B**) A liner calibration standard curve (particle number vs. count). The counts of particles with the pre-set numbers were measured using DLS, followed by generation of a linear calibration curve (R^2^ = 0.9978). For A and B, data representative of two independent experiments. (**C–E**) MVs were isolated from the Beas2B cells after hyperoxia. Total MV number (**C**) total MV protein amount (**D**) and protein amount per MV (total MV protein/MV counting numbers) (**E**) were shown. (**F–H**) Large and small RNAs were purified from the isolated MVs and subjected to electrophoresis using agarose gel, as illustrated in (**F**). Total RNA amount is shown in (**G**). RNA amount per MV (total MV RNA/MV counting numbers) are shown in (**H**). For **C–H**, data represent mean ± SD of three independent experiments.

To better quantify the amount of MVs induced by oxidative stress, we first established a method to quantify the MV counts. Initially, MVs were fluorescently labeled with protein-carboxy-fluorescein-succinimidyl ester (CFSE) as previously described^36^, verifying that vesicle numbers were increased in a dose-dependent manner (Fig. 3A). A standard curve between the pre-set particle numbers and the measured particle counts using DLS was set as detailed in the Material and Methods (Fig. 3B). Therefore, the MV numbers in a given sample can be indirectly calculated based on the DLS counts using the standard curve. Using this method, we assessed the MV numbers in the absence and presence of hyperoxia (Fig. 3C). Total MV protein amount is shown in Fig. 3D. Next, we determined the amount of MV protein per each MV (Fig. 3E). Interestingly, the same amount of protein was found in the MVs generated in the absence or presence of hyperoxia (Fig. 3E). After hyperoxia, the MV RNAs were subjected to agarose gel fraction (Fig. 3F). Based on their number of base pairs, small size and large size of MV-RNAs after RA or hyperoxia are shown in Fig. 3G. After normalized with the MV counts, we found that in a given MV, RNAs with smaller sizes were increased much more robustly compared with RNAs with larger number of base pairs (Fig. 3H). This observation prompted us to focus on MV-miRNAs which only have approximately 20–22 base pairs of nucleotides.

### Hyperoxia-induced, epithelial cell-derived MV promotes immunomodulatory cell recruitment *in vivo* and macrophage activation *in vitro*

**Figure 4:**
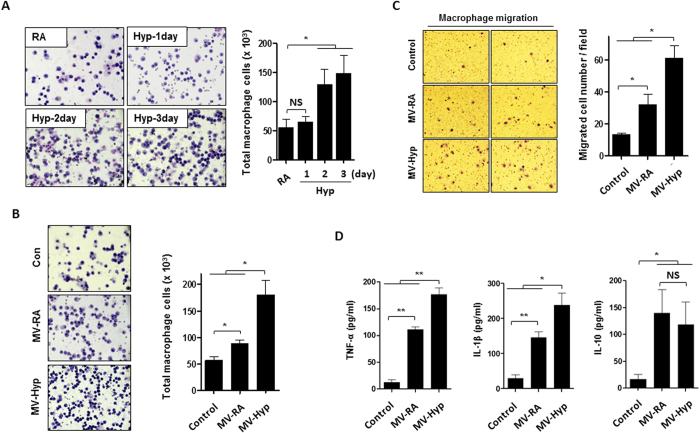
Lung epithelial cell-derived MVs induce macrophage migration and cytokine release. (**A**) Mice were exposed to hyperoxia for the designated time period. BALF cells were stained with hematoxylin and eosin (H&E), followed by cell counting. n = 4 mice per group. (**B**) BALF-derived MVs were isolated from mice 3 days after hyperoxia. BALF cells were stained with H&E and analyzed 24 h after exposed to the RA-MVs and hyperoxia-induced MVs (10 μg/50 μl per mice) intranasally. n = 4 mice per group. (**C**) MVs were isolated from the Beas2B cells 2 days after hyperoxia. THP1 macrophages were treated with the Beas2B cell-derived MVs (5 μg/500 μl per well). After 16 h, transwell migration assay of THP1 macrophages was performed, as described in Material and Methods. Representative images of migrated cells (left panel) and quantification graphs (right panel) are shown. (**D**) THP1 macrophage were treated with isolated MVs (5 μg/500 μl per well) described in (**B**). After 16 h, TNF-α, IL-1β, and IL-10 were analyzed using ELISA. For C and D, data represent mean ± SEM of three independent experiments.

Macrophage cell counts were increased in BALF obtained from the mice that were exposed to hyperoxia in a time-dependent manner (Fig. 4A), compared to those that were exposed to RA. We isolated the hyperoxia-induced MVs from BALF and treated the C57BL/6 mice *via* inhalation. As shown in Fig. 4B, inhaled MVs significantly enhanced the macrophage cell counts in BALF, particularly in the presence of hyperoxia (Fig. 4B). We also isolated the hyperoxia-induced, human epithelial cell (Beas2B) -derived MVs and treated the human THP1 macrophage *in vitro* using these isolated MV. Macrophage migration was markedly stimulated after being treated with the Beas2B-derived MVs *in vitro* (Fig. 4C). Furthermore, TNF-α, IL-1β and IL-10 secretions from THP1 macrophages were measured. Significantly enhanced TNF-α and IL-1β secretions after exposure to hyperoxic MVs were observed (Fig. 4D, left and middle panels).

### Hyperoxia augments specific miRNA levels in MVs and the hyperoxia-upregulated miRNAs activate macrophages in a synergistic manner

**Figure 5:**
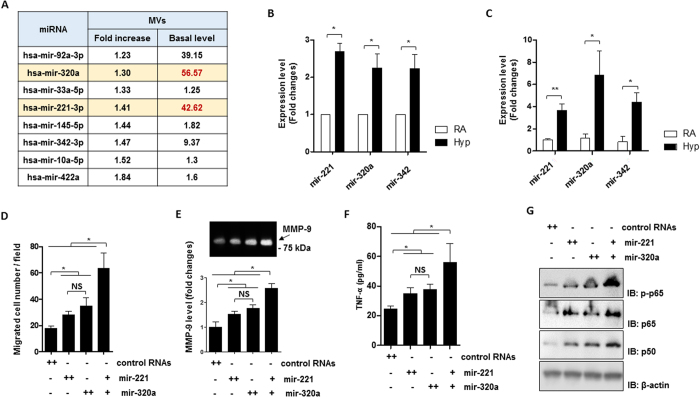
BALF- and lung epithelial cell-derived MVs contain pro-inflammatory miRNAs. (**A**) Elevated miRNAs derived from BALF MVs after hyperoxia. The table was generated from miRNA microarray data as shown in Fig. 2A. (**B,C**) MVs were isolated from Beas2B cells (**B**) and primary epithelial cells (**C**). RNA was isolated from the MVs and quantified using the real-time quantitative RCR (qPCR). Individual miRNA expression levels were shown in bar graphs (**D**). Transwell migration assay of THP1 macrophages was performed after treated with mir-221 and/or mir-320a mimics which were transfected using lipofectamine 2000, as described in Material and Methods. (**E–G**) THP1 macrophages were treated with mir-221 and/or mir-320a mimics. After 16 h, MMP-9 levels (**E**) and TNF-α levels (**F**) from the culture medium were analyzed using gelatin zymography and ELISA, respectively. Western blot analysis was performed using total cell lysates with the indicated antibodies (**G**). For (**B–F**) data represent mean ± SD of three (**D–F**) or four (**B,C**) independent experiments. For (**G**) data represent two independent experiments with the similar results. Unprocessed original scans of gel and blots are shown in Suppl. Fig. 1.

We next determined the miRNA compositions in hyperoxic MVs obtained from BALF after hyperoxia. A number of upregulated miRNAs were identified in hyperoxic MVs using miRNA profiling, as described in Materials and Methods. Figure 5A shows 8 miRNAs whose levels were significantly elevated in BALF MVs after hyperoxia (Fig. 5A). We chose three specific miRNAs (miR-320a, miR-221, and miR-342) and further confirmed their enhanced expression in Beas2B epithelial MVs after hyperoxia (Fig. 5B). Similar observations were found using mouse lung primary epithelial cells (Fig. 5C). To illustrate the functions of these hyperoxia-induced miRNAs, we transfected THP1 macrophages with miR-221 and/or miR-320a mimics. Macrophage migration assays were performed as described in Materials and Methods. Both miR-221 and miR-320a promoted macrophage migration significantly. More interestingly, miR-221 and miR-320a robustly exerted synergistic effects on macrophage migration (Fig. 5D). We further found that miR-221 and miR-320a up-regulated MMP9 (Fig. 5E), a molecule which is known to be involved in macrophage migration^37^,^38^,^39^. Similarly, a synergistic effect between miR-221 and miR-320a on macrophage-derived TNF-α secretion was also observed (Fig. 5F), in addition to miR-221 or miR-320a inducing TNF-α secretion individually (Fig. 5F). Consistently, the well-known mechanistic pathway NF-κB pathway components were targeted and up-regulated by miR-221 and miR320a (Fig. 5G).

### MVs activate macrophages via MV-shuttling miRNAs

**Figure 6:**
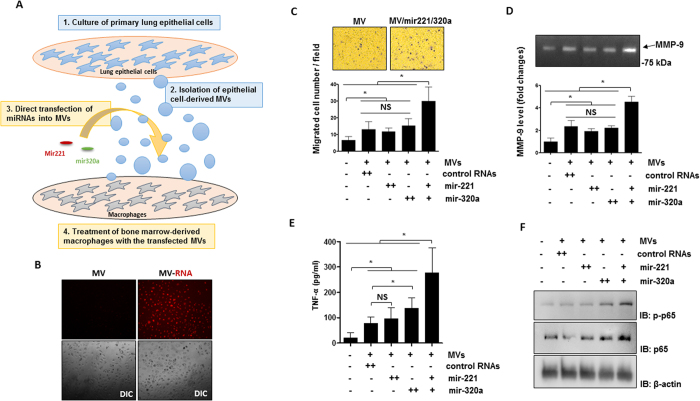
Synergistic effects of MV-containing mir-221 and 320a on the macrophage migration and cytokine secretion. (**A**) Schematic illustration of the functional analysis of MV-containing miRNAs on the macrophage activation. MVs were isolated from the mouse primary lung epithelial cells and directly transfected with mir-221 and/or 320a as described in Material and Methods. Mouse BMDMs were treated with the transfected MVs and the macrophage migration and cytokine secretion were measured. (**B**) MV-containing RNAs derived from the primary epithelial cells were labeled with acridine orange and incubated with the mouse BMDMs. After 16 h, MV-RNAs were visualized using fluorescent microscope. The MV internalization was shown in the upper panels. Differential Interference Contrast (DIC) cell images were shown in the lower panels. (**C–F**) Mouse BMDMs were treated with the transfected MVs (5 μg/500 μl per well) described in (**A**). After 16 h, BMDM migration was measured using the transwell assay, as described in Material and Method (**C**). Representative images of migrated cells (upper panel) and quantification graphs (lower panel) are shown. MMP-9 levels (**D**) and TNF-α levels (**E**) from the culture medium were analyzed using gelatin zymography and ELISA, respectively. Western blot analysis was performed using total cell lysates (**F**) with the indicated antibodies. For (**C–E**) data represent mean ± SD of three independent experiments. For (**B,F**) data represent two independent experiments with the similar results. Unprocessed original scans of gel and blots are shown in Suppl. Fig. 1.

In Fig. 5, we demonstrated that hyperoxia augmented certain specific miRNAs in the epithelial cell-derived MVs. Furthermore, these miRNAs synergistically activated macrophages. We next ask whether these “pro-inflammatory” miRNAs can be delivered to the recipient cells in a MV-shuttling manner. We isolated and cultured the mouse primary lung epithelial cells. MVs were isolated from these cells and selected miRNAs were directly transfected into isolated MVs as described in Materials and Methods. Here we chose miR-221 and miR-320a as examples to be consistent with the above data. MVs with enhanced miR-221 and/or miR-320a were used to treat mouse BMDMs, as illustrated in Fig. 6A. We first confirmed that MV-shuttling RNAs were successfully delivered into the recipient cells using labeled MV-RNAs (Fig. 6B), as described in the Materials and Methods. BMDMs treated with MVs with the enhanced miR-221 and/or miR-320a augmented BMDM migrations. More importantly, MVs which contain both the enhanced miR-221 and miR-320a carried the strongest effects on promoting macrophage migration (Fig. 6C). TNF-α secretion and MMP-9 level/activity were also enhanced in the same pattern, i.e. MV-miR-221 and MV-miR-320a synergistically up-regulated MMP level and activities, as well as TNF-α secretion from BMDMs (Fig. 6D,E, respectively). Consistent results were found on the expression of NF-κB pathway components (Fig. 6F).

## Discussions

Emerging evidence suggests that EVs potentially can serve as novel targets for the development of therapeutic and diagnostic reagents. While intensive studies have focused on the role of EVs in cancer metastasis, the generation, regulation and function of EVs in lung diseases remain largely unexplored. EVs describe a group of heterogeneous vesicles which have different, characteristics, mechanisms of formation and functions^5^,^19^,^22^. To develop novel therapeutic agents and diagnostic markers, the types of EVs and the compositions of EVs, along with their dynamic changes in the pathogenesis of lung diseases, require a complete characterization. Our reports, for the first time, demonstrated that after oxidative stress, MVs, rather than exosomes, are the main types of EVs that are generated by live lung epithelial cells.

Small RNAs are the main MV-components that change the most significantly when compared with proteins or large RNAs during hyperoxia-associated oxidative stress (Fig. 3H).

Our data further identified the MV-miRNA profiles which have been significantly altered after hyperoxia (Fig. 2A). Using realtime PCR, we confirmed that a number of miRNAs robustly was increased in the epithelial cell-derived MVs after exposure to hyperoxia (Fig. 5B,C). Interestingly, despite the rapidly growing interests that have focused on the potentials of EV-miRNAs to serve as biomarkers or therapeutic targets, the number of copies of “highly upregulated” miRNAs found in tumor cells remains at a very low level in each individual exosome detected in the plasma^34^. Therefore, whether the miRNAs are the main functional components in exosomes is ambiguous^23^,^40^. Our studies show that after hyperoxia, the main form of epithelial cell-derived EVs is MVs rather than exosomes. First of all, a relatively high copy numbers of MV-miRNAs were induced by hyperoxia (Fig. 5B,C). More importantly, a group of miRNAs, but not only one single miRNA, were upregulated in MVs (Figs 2A and 5A). Our functional studies confirmed that many of these upregulated MV-miRNAs carried functional effects and promoted TNF-α and IL-1β secretions from macrophages (Fig. 6C–F). Robust synergistic effects were observed among the upregulated MV-miRNAs, suggesting crucial roles of epithelial cell-derived MV-miRNAs in mediating macrophage activation. Our studies illustrate the complexity of developing diagnostic/therapeutic targets using EV or EV compositions. First, the pattern of different type of EVs involved in the pathogenesis requires a detailed characterization. This process is probably stimulation-dependent and cell type-dependent. Second, multiple compositions in certain type of EVs involved in the disease process needs to be delineated. Third but not the last, the synergistic effects of different EV compositions are to be addressed.

The underlying mechanisms to generate MVs and exosomes are clearly different. MVs are formed *via* direct protruding of cell plasma membrane while exosomes are much smaller vesicles which are generated after multivesicular bodies (MVBs) fuse with the plasma membrane^20^. GW182 and AGO2, two main components of the RNA-induced silencing complex (RISC) that congregate with endosomes and MVBs^41^. Exosomes have been shown to contain GW182 but not AGO2^41^. Therefore, GW182-associated miRNAs are more likely being found in exosomes, possibly in association with only certain types of disease process.

In addition to miRNAs, the content of MVs and exosomes is highly enriched with a variety of other components, including the proteins derived from the parent cells^21^,^42^. However, it is unclear whether all of these components indeed carry significant biological functions or simply represent debris from cell damage. Previously, we have shown that certain protein components in the epithelial cell-derived EVs can function as a signal transmitter, such as through caspase-3^14^. In the previous report, we did not clarify which type of EVs consists of caspase-3. Based on the sizes, presumably, the caspase-3 also resides in epithelial cell-derived MVs. Exosomal protein is likely at relatively smaller size and consists of more fragments of proteins.

Our data suggest that the number of small RNAs including miRNAs increased more dramatically after hyperoxia, compared with large RNAs and proteins; therefore, this report did not specifically explore the potential functions of MV-proteins. Furthermore, this current study did not consider the effects of MVs on macrophage activation *via* cell surface receptors. Instead of engulfing all the MV compositions, macrophage activation potentially can be triggered *via* the formation of macrophage surface receptor-MV complex, followed by a signaling cascade to activate macrophages. Detailed characterizations of the proteins presented on the surface of epithelial cell-derived MVs after oxidative stress require further exploration. Another remaining limitation involving our studies is the difficutly in determining the minimum amount of MVs or MV-miRNAs that are required to activate the recipient macrophages, particularly *in vivo*. Presumably, this question is largely affected by how many different miRNAs and proteins are altered by each stimulation. Therefore, this minimum “dose” of MV-miRNAs may also be cell type and stimulation dependent.

In summary, our report finds that after hyperoxia-associated oxidative stress, live lung epithelial cells release a significant amount of MVs, rather than Exos or ABs. Abs will be increased after prolonged exposure. MiRNAs are the main MV components which are altered robustly in each individual MV after hyperoxia. Multiple MV-miRNAs activate macrophages and subsequently exert pro-inflammatory effects, in a synergistic manner.

## Materials and Methods

### Materials

MiRNA-221 mimics (HMI0398), miRNA-320a mimics (HMI0470), miRNA-92a mimics (HMI0955), and miRNA-422a (HMI0562) were purchased from Sigma Aldrich (St. Louis, MO). Rabbit anti-phospho-p65 (Ser536) (3033), anti-p65 (8242) and anti-p50 (3035) were purchased from Cell Signaling (Danvers, MA). Human TNF-alpha DuoSet ELISA (DY210), Human IL-1 beta/IL-1F2 DuoSet ELISA (DY201), Human IL-10 DuoSet ELISA (DY217B), and Mouse TNF-alpha DuoSet ELISA (DY410) were purchased from R&D system (Minneapolis, MN).

### Animals

Wild type C57BL/6 mice (male, 6 to 8 weeks of age) were obtained from Charles River Laboratories (Wilmington, MA, USA). All the protocols involving animals in this study were approved by the institutional animal care and use committee (IACUC) of Boston University. All experimental protocols and methods were approved by Boston University and were carried out in accordance with the approved guidelines.

### Mouse primary cell isolation

Primary alveolar epithelial cells were isolated from mice as described previously^43^. Briefly, mouse lung tissue was washed with sterile PBS, followed by infusion of 2 ml dispase and 0.5 ml 1% agarose. Lung tissue was then dissociated in DMEM with 25 mM HEPES and 200 U/ml DNase. Isolated cells were sequentially passed through cell strainer (using 100 μm, 40 μm and 20 μm cell strainer), followed by incubation on the plates pre-coated with CD45 and CD16/32 antibodies for 2 h. Suspended cells were further transferred to non-coated plates to get rid of fibroblast. After another 2 h incubation, suspended epithelial cells were culture in DMEM containing 10% FBS and subjected to further experiments.

Bone marrow-derived macrophages were isolated from mice as described previously^44^. Isolated bone marrow cells were cultured with 30% of L929-conditioned medium for 5 to 7 days to allow differentiation of the bone marrow monocyte/macrophage progenitors, and subjected to further experiments.

### Cell culture

Human Beas2B lung epithelial cells and human THP1 monocytes were obtained from American Type Culture Collection (ATCC) and maintained in Dulbecco’s modified Eagle’s medium (DMEM), or RPMI-1640 with 10% fetal bovine serum (FBS) and 1% penicillin/streptomycin. Cells were cultured at 37 °C in a humidified atmosphere with 5% CO_2_ and 95% air. For hyperoxia exposure, cells were exposed to hyperoxia (95% oxygen with 5% CO_2_) in modular exposure chambers, as previously described^45^.

### Categorize the EVs

Three types of EVs were prepared by using sequential centrifugation protocols described previously with a minor modification^23^,^46^. Cultured cells were incubated with culture medium containing EV-depleted FBS for desired time points. Conditioned medium was collected and centrifuged at 300 g for 10 min to remove floating cells. The supernatant was further centrifuged at 2000 g for 20 min to pellet ABs. To isolate MVs, the AB-depleted supernatant was passed through a 0.8-μm-pore filter followed by centrifugation at 16,000 g for 40 min. Finally, the resulting supernatant was passed through a 0.2-μm-pore filter and ultracentrifuged at 100,000 g for 1 h to pellet Exos. Each vesicles were further washed with cold PBS, then re-suspended with PBS and stored at −80 °C. Protein concentration was measured with a Bradford assay.

### RNA preparation, reverse transcription, and quantitative real-time PCR

Total RNAs were purified from isolated EVs using MiRNeasy Mini Kits (cat. no. 217004; Qiagen, Valencia, CA). Purified RNA amount was measured by NanoDrop Lite Spectrophotometer (Thermo Scientific). Reverse Transcription Kit (cat. no. 4374966, Thermo Fisher Scientific) was used to generate single stranded cDNA from the equal amount of purified RNAs. SYBR green-based real-time quantitative PCR (qPCR) technique was performed for detection of miRNAs as previously described^47^,^48^. The list of primers is shown in Table 1.

### Electron microscopy

Purified EVs were fixed on the formvar-carbon coated mesh 400 grid according to the manufacturer’s recommendation (101Bio, Palo Alto, CA). EV were visualized using transmission electron microscopy (Experimental Pathology Laboratory Core, Boston University School of Medicine).

### Dynamic Light Scattering analysis

Size of EVs were analyzed using dynamic light scattering (DLS) instrument (Brookhaven 90plus Nano-particle Sizer, Biomedical Engineering core, Boston University). For calculating absolute EV number, standard calibration curve (particle number vs. count rate) was generated using DLS (R^2^ = 0.9978). Count rate of each EV samples were measured by DLS, followed by calculation of absolute vesicle number.

### Quantitative analysis of MV contents

Total MV number, protein, and RNA amount (small and large RNAs) were obtained under RA and hyperoxia condition respectively. Next, we calculated the mean values of protein and RNA amount in single MV by dividing with MV counting number.

### MiRNA microarrays

Three types of EVs were isolated from mouse BALF, followed by purification of total RNAs. The prepared RNA samples were analyzed with Multiplex miRNA Assay Immunology Panel (Firefly, ab204064, abcam). Microarray heatmap was generated using Firefly^®^ Analysis Workbench software.

### MiRNA transfection into MVs

Isolated MVs were incubated with Exo-Fect reagent [company?] and miRNA mimics (10 pM) at 37 °C for 10 min and placed on ice for 30 min, followed by three times washing with PBS using 16,000 g-force centrifugation. Successful direct-transfection was confirmed using qPCR (not shown). The transfected MVs were directly used for further experiment.

### Transwell migration assay

Macrophage migration analysis were performed using 6.5-mm-diameter polycarbonate 8-m microporous membranes (Costar, Cambridge, MA), as described previously^49^. 3 × 10^4^ of the macrophages were placed on the inner chamber. EV-depleted 10% FBS was added in the outer well with miRNA-transfected MVs or miRNAs wrapped by lipofectamine 2000 (11668027, Thermo Fisher Scientific). After 16 h incubation, migrated cells were fixed with 4% formaldehyde and stained with hematoxylin. Migrated cells were counted using microscope.

### ELISA assays

Macrophages were treated with miRNA-transfected MVs or miRNAs wrapped by lipofectamine 2000 for 16 h, followed by collection of conditioned medium. TNF-α, IL1β, and IL10 cytokine levels were analyzed using DuoSet^®^ ELISA Development Systems (R&D system), according to the manufacturer’s recommendation.

### Gelatin Zymography

Prepared samples were loaded onto a SDS-PAGE containing 1 mg/ml gelatin and separated by electrophoresis under non-reducing condition. After washing with a 2.5% Triton X-100 for 1 h, the gel was incubated in developing buffer containing 50 mM Tris-HCl (pH 7.5) and 5 mM CaCl_2_ at 37 °C for 16 h. Gelatin lytic activity was analyzed by staining with Coomassie Brilliant Blue G-250.

### Western Blot Analysis

Harvested cells were lysed with RIPA buffer containing 1% triton X-100, protease inhibitor, and phosphatase inhibitor. Western blotting analysis was performed as described previously^50^.

### Statistical analysis

All experiments were independently repeated 2–4 times. Represented data of the identical results were shown in the presented figures. The exact n values of each experiments and statistical significances were shown in the corresponding figure legends. Statistical analysis was performed with unpaired two-tailed Student’s T-test. Values of p < 0.05 were considered statistically significant (*p < 0.05, **p < 0.01).

## Additional Information

**How to cite this article**: Lee, H. *et al*. Epithelial cell-derived microvesicles activate macrophages and promote inflammation *via* microvesicle-containing microRNAs. *Sci. Rep*. **6**, 35250; doi: 10.1038/srep35250 (2016).

## Supplementary Material

Supplementary Information

## Figures and Tables

**Table 1:** Sequences of primers used in qPCR.

Name	primer sequence for RT (5′ to 3′)	primer sequence for qPCR (5′ to 3′)
Universal primer		GGTGTCGTGGAGTCGGCAATTCAGTTGAG
miR-221	CTCAACTGGTGTCGTGGAGTCGGCAATTCAGTTGAGGAAACCCA	ACACTCCAGCTGGGAGCTACATTGTCTGCT
miR-320a	CTCAACTGGTGTCGTGGAGTCGGCAATTCAGTTGAGTCGCCCTC	ACACTCCAGCTGGGAAAAGCTGGGTTGAG
miR-342	CTCAACTGGTGTCGTGGAGTCGGCAATTCAGTTGAGACGGGTGC	ACACTCCAGCTGGGTCTCACACAGAAATCG
miR-92a	CTCAACTGGTGTCGTGGAGTCGGCAATTCAGTTGAGACAGGCCG	ACACTCCAGCTGGGTATTGCACTTGTCCC
miR-422a	CTCAACTGGTGTCGTGGAGTCGGCAATTCAGTTGAGGCCTTCTG	ACACTCCAGCTGGGACTGGACTTAGGGTCA
