# Supplementary material for: Epithelial cell-derived microvesicles activate macrophages and promote inflammation via microvesicle-containing microRNAs
Source: Sci Rep. 2016 Oct 12;6:35250. doi: 10.1038/srep35250 (PMC5059671; doi:10.1038/srep35250)
Supplement: Supplementary Information [file srep35250-s1.pdf]

Epithelial cell-derived microvesicles activate macrophages and promote inflammation *via* microvesicle-containing microRNAs

Heedoo Lee[#1], Duo Zhang[#1], Ziwen Zhu[1], Charles S. Dela Cruz[2] and Yang Jin*[1]

[1]Division of Pulmonary and Critical Care Medicine, Department of Medicine, Boston University, Boston, MA 02118

[2] Section of Pulmonary, Critical Care and Sleep Medicine, Yale University School of Medicine, New Haven, CT 06519

[#] These authors contributed equally to this work

*Address correspondence to Yang Jin MD, Ph.D., Division of Pulmonary and Critical Care, Department of Medicine, Boston University Medical Campus, 72 East Concord St., Boston, MA 02118

yjin1@bu.edu

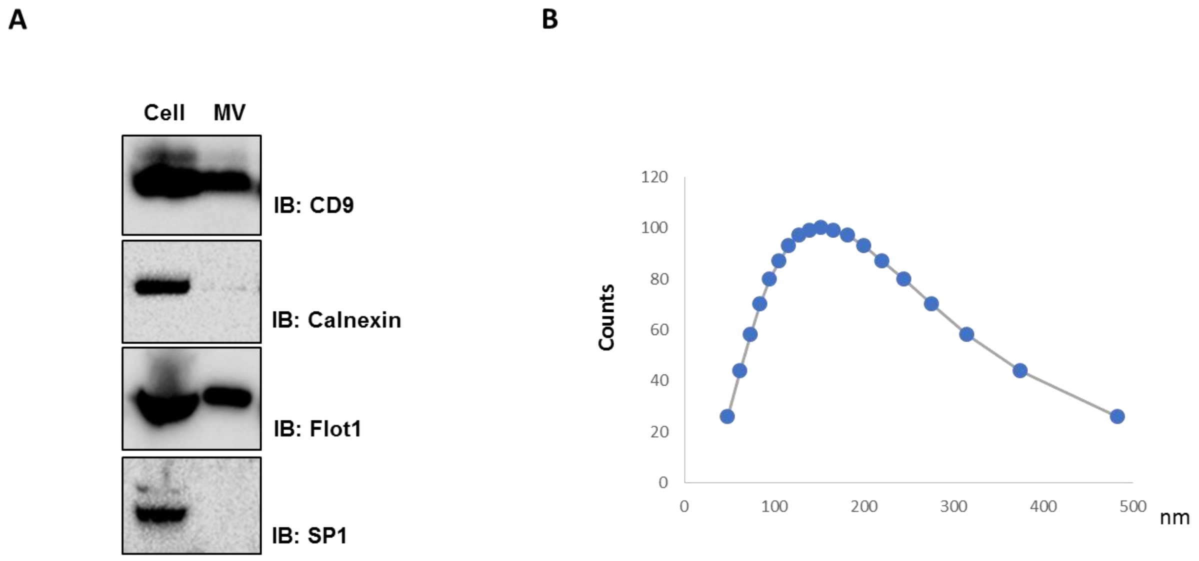

Supplementary Figure 1. Verification of MVs derived from lung epithelial cells. (A-B) MVs were isolated from Beas2B cells. Western blot analysis was performed using total cell lysates and the isolated MVs with the indicated antibodies (A). The size distribution of the isolated MVs was analyzed using Dynamic Light Scattering (B).

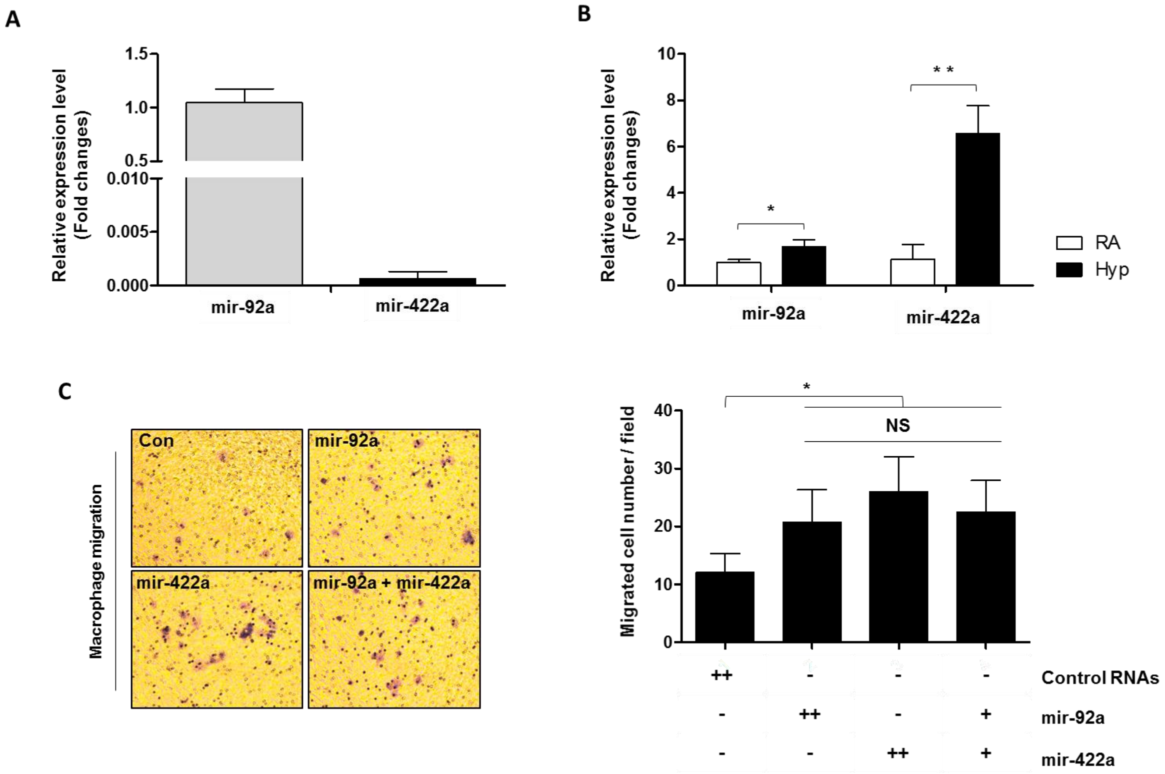

Supplementary Figure 2. Expression of mir-92a and 422a in lung epithelial MVs and their effects on the macrophage migration. (A-B) MVs were isolated from Beas2B cells. RNA was purified from the isolated MVs and quantified using the real-time quantitative RCR (qPCR). Relative expression levels of miRNAs (A: miR-92a vs. miR-422a and B: RA vs. Hyperoxia) were shown in bar graphs. (C) Transwell migration assay of THP1 macrophages was performed with mir-92a and/or mir-422a mimics, as described in Material and Methods. Data represent mean ± SD of three (A and B) or four (C) independent experiments.

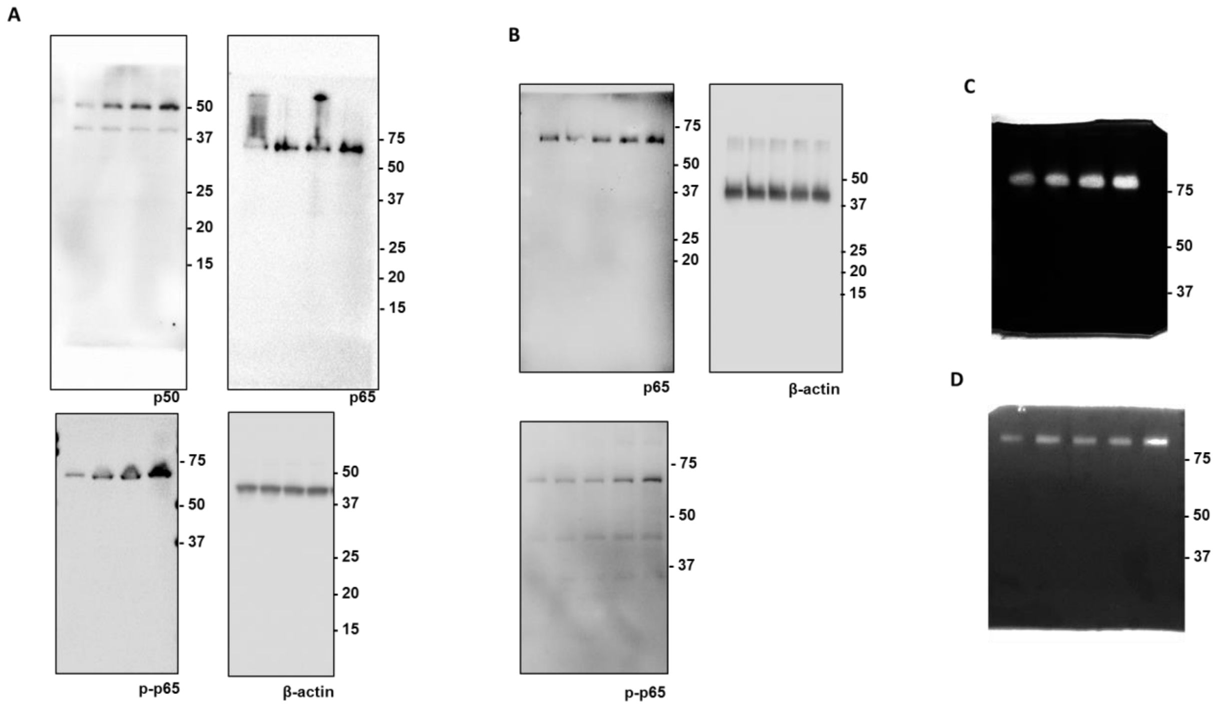

Supplementary Figure 3. Uncropped images of the western blotting and zymography results.
